# Health Information Discrepancies Between Internet Media and Scientific Papers Reporting on Omega-3 Supplement Research: Comparative Analysis

**DOI:** 10.2196/ijmr.8981

**Published:** 2018-10-01

**Authors:** Daryl Nault, Ariel Beccia, Haruka Ito, Sarah Kashdan, Angela Senders

**Affiliations:** 1 Helfgott Research Institute School of Research and Graduate Studies National University of Natural Medicine Portland, OR United States; 2 Research Department Maryland University of Integrative Health Laurel, MD United States; 3 Quantitative Health Sciences University of Massachusetts Medical School Worcester, MA United States

**Keywords:** consumer health information, health literacy, health communication, health promotion, evidence-based practice, dietary supplements, omega-3 fatty acids, journalism

## Abstract

**Background:**

Dietary supplements are the most used complementary and alternative health modality in the United States, and omega-3 supplements continue to be the most popularly used nonvitamin or nonmineral supplements by adults. Users of dietary supplements report that they obtain health guidance from internet media resources, but there is question as to whether or not these resources provide the necessary evidence to guide health decisions. Current evidence suggests that there is a mistranslation occurring somewhere between researchers and the media.

**Objective:**

The aim of this study was to conduct a comparative cross-sectional analysis to identify areas of discordance created when science is translated from the laboratory to Web-based news media.

**Methods:**

A Google news search provided our convenience sample of 40 omega-3 supplement–based media reports stratified by the years 2009 to 2012. Media reports (n=17) were compared with the corresponding scientific papers for content. Report and scientific paper content were extracted using commonly accepted reporting guideline domains, and domains were then compared for detecting underlying omissions or mistranslations in reporting. Mean scores for all of the scientific papers and media reports were assessed for each domain.

**Results:**

Scientific papers (n=14) generally maintained a mean close to complete for each reporting domain. The only domain where there was not a significant difference between media and scientific reporting match was within the objectives domain (χ^2^_1_*=* 0.8, *P*=.36). Media reports (n=17) more frequently reported potential caveats and warnings for consumers with a mean domain for caveat reporting of 0.88, with possible scores falling between 0 and 1.

**Conclusions:**

There are inherent differences in the intended audience, structure, and goals in scientific and media communications. These differences should be explored further, and consumers should be made aware of them. Additional considerations for balanced reporting and reader accessibility are also necessary to take into account and are explored further in this analysis.

## Introduction

### Background

More than half of the US adults take dietary supplements, making supplementation the most used complementary and alternative health modality [1-5]. Since 2007, omega-3s have been the most used nonvitamin and nonmineral dietary supplement according to the National Health Interview Survey (NHIS) data [1,6]. In one large survey, 40% to 45% of respondents said that they had used internet and news media as resources to obtain dietary supplement information [5]. Conversely, less than a quarter (23%) of respondents using dietary supplements on the most recent NHIS Alternative Health Survey reported that their use was because of the advice of a health care professional [6]. What is possibly more concerning is that about 25% of these same users of dietary supplement had not disclosed their use of supplement to their doctor [7]. Patients attempting to improve their health through the use of dietary supplements may have the best intentions in mind, but they may potentially be causing more harm than good. For this reason, it is important to identify the information obtained from Web-based resources so that we might improve the ways we inform these proactive health information consumers.

Despite how frequently their advice is followed, Web-based news resources may contain inaccurate information. It has been suggested in the past that Web-based health information can be misleading or exaggerated, especially when compared with scientific evidence [8-13]. These mistranslations may be more likely to occur between health research and media reporting [10-13]. This identifies *what* the problem is, but there is no current definitive answer to *where* these mistranslations may occur. Therefore, we sought to investigate potential discrepancies between publicly available Web-based health information (Web media reports) and the corresponding scientific evidence upon which those reports were based. Using a ubiquitous search engine fitted with specific parameters, we identified and extracted scientific evidence presented in Web-based media reports of omega-3 supplement research. Media-reported information was then compared with the scientific paper that was referenced. An analysis of the content provided in each resource will hopefully clarify where scientific and media dissemination differences occur. These findings may serve to better educate consumers of the health information on the information they may miss out on, especially when they do not fully communicate decisions with health care professionals.

### Omega-3 Supplement Reports From 2009 to 2012

We chose to focus on a commonly used supplement during the time when use was most prevalent. The number of peer-reviewed scientific papers on omega-3 supplements began to rise in the early 2000s [14]. With an increase in omega-3 research, there was a corresponding rise in public interest. Between 2007 and 2012, popularity of use of omega-3 supplement nearly doubled (23% increase), whereas other supplements saw little to no increases in use during the same period [1]. The public has also expressed a growing interest in finding information about omega-3s. In early 2010, Google term-frequency reports show searches for *fish oil* and *omega-3* increased up until their peak popularity [15]. To capture a sample of media reports that were most likely influential, we limited our search to news media reports on omega-3 supplements posted between 2009 and 2012.

### Objectives

The descriptive objective of this comparative analysis was to summarize how omega-3 supplement guidance was provided through Web-based news media (2009-2012). Our comparative objective was to identify where reporting gaps occurred between applicable media reports and corresponding scientific publications. As exploratory objectives, we also took balance of reporting, readability, and accessibility into account to demonstrate additional factors that might impact comprehension and decision making of a health information consumer.

## Methods

### Study Design

One author (DN) performed the Google news search and assessed inclusion criteria for Web-based health blogs and news stories on omega-3 dietary supplements, hereafter referred to as *media reports* with agreement from AS. Individual search pages were saved and archived for future use and transparency. Four authors (AB, HI, SK, and DN) independently extracted data in duplicate from media reports, using a standardized data collection form. Before data extraction, authors met for a brief training session on using the data collection form to improve the consistency of data collection. Next, the same authors independently extracted data in duplicate from each of the corresponding omega-3 dietary supplement scientific papers referenced by the media reports. Finally, DN compared the data extracted from media reports and scientific papers to assess match of information between media and corresponding scientific resources.

### Search Strategy for Identification of Media Reports

**Figure 1:**
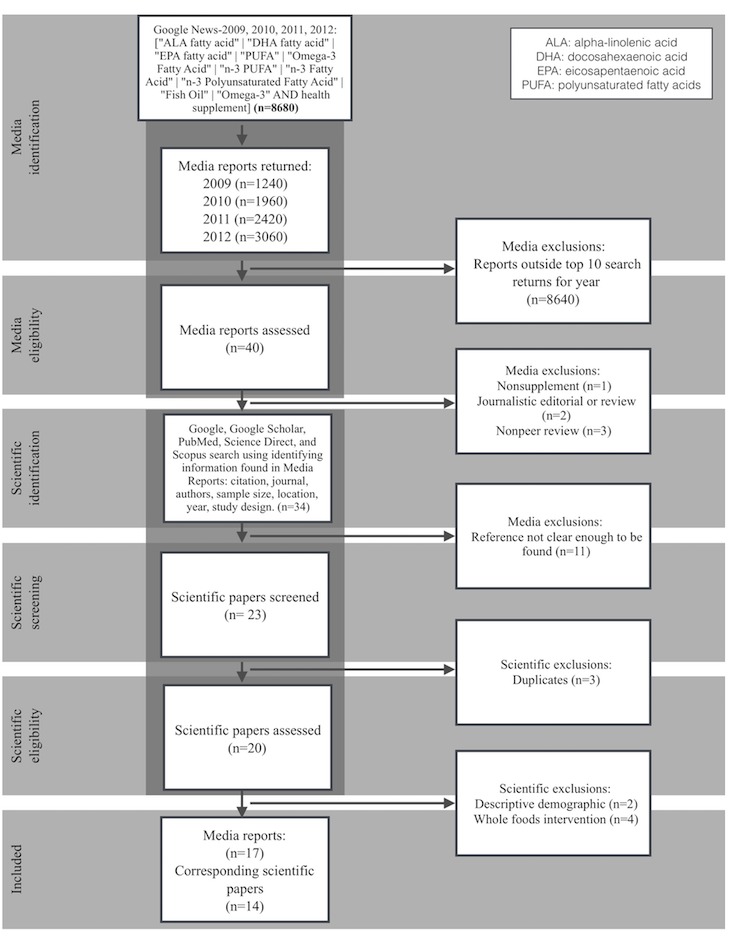
Media report and corresponding scientific paper search and screening flow diagram. Media reports were initially excluded on the basis that they did not refer to dietary supplements specifically, were editorials or reviews, were based on nonpeer-reviewed evidence, or if their referencing information was not clear enough to link back to a specific scientific paper. Scientific papers were excluded if they were duplicates, they reported solely on dietary supplement use demographic statistics, or were only based on dietary interventions.

We searched Google News between January 1 and December 31 for each year from 2009 to 2012, inclusive (Figure 1). This search was performed through an incognito window, without a user log-in, and in a newly downloaded Google Chrome app. All search return pages were archived to preserve the exact returns given at the time they were searched (December 2015). The search query was run once in Google News, and filters were then used to restrict dates of publication. Google PageRank has changed since its inception, but the basic idea behind Google search ranking remains the same: returns are ranked higher based on keyword relevancy and page popularity, as measured by user interest and recurring links [16]. Prior evidence suggests that the first 10 (or first page of) search returns are typically the most viewed, in some cases, receiving close to 100% of link traffic [17-23], and are therefore most likely to have the highest viewership for any particular search. Thus, we used a convenience sample consisting of the top 10 search returns for each year (2009-2012), totaling 40 media reports to be assessed for inclusion criteria.

The search query used to obtain relevant media reports was as follows: [“ALA fatty acid” OR “DHA fatty acid” OR “EPA fatty acid” OR “PUFA” OR “Omega-3 Fatty Acid” OR “n-3 PUFA” OR “n-3 Fatty Acid” OR “n-3 Polyunsaturated Fatty Acid” OR “Fish Oil” OR “Omega-3” AND health supplement]. This search query was determined by both the terminology used in the NHIS to define these health supplements: “Fish oil (or omega-3, DHA, or EPA fatty acid)” and the use of PubMed medical subject headings to find additional descriptors for this class of supplements such as polyunsaturated fatty acids (PUFA), n-3, and alpha-linolenic acid (ALA).

We included health blogs and news reports that were published on Web, in English, and fell within the top-10 returns for each year between January 1, 2009 and December 31, 2012. Media reports about omega-3 supplements that referenced a peer-reviewed scientific paper were included. Media reports that were simply *expert opinion* or *reviews not published in peer-reviewed literature* of omega-3 evidence were excluded, as they did not address 1 primary scientific research publication and therefore would be inappropriate for this appraisal method.

### Search Strategy for Identification of Scientific Papers

Any information found within a media report that might identify the corresponding scientific paper was used to locate the full-text manuscript through Google, Google Scholar, PubMed, ScienceDirect, and Scopus. This information included but was not limited to any part or combination of the paper: full citation, author names, journal name, year of publication, sample sizes, location of the study, and study design.

We included papers that reported on controlled and uncontrolled intervention studies, observational studies, and narrative reviews of intervention studies (Figure 1). Both animal and human studies regarding the interventions of omega-3 dietary supplement were included. Cross-sectional reports on use of supplement *descriptive demographics* were excluded. Studies that aimed to alter or quantify a participant’s *whole food* dietary consumption of omega-3s were also excluded. There were no restrictions on type or outcome measure of participants, as our purpose was to assess how outcomes were presented to the public through media reports, independent of the outcome type.

### Data Extraction

We developed a standardized data extraction form using the EQUATOR Network’s guidelines for research reporting (Multimedia Appendix 1; [24]). The following domains were extracted: study objectives, design, study population characteristics, intervention or exposure, comparison or control, outcome measure, participant attrition, statistical analyses, limitations, caveats (or warnings for consumer or clinical use), and results. The following data points were also collected: date of posting, URL, title, and author names. Two review authors independently extracted data from each media report and scientific paper. We used the same data extraction form for both resource types; disagreements were discussed by the authorship team and resolved by consensus.

### Primary Analysis

Scientific papers were assessed first, according to whether or not they provided the relevant information from the EQUATOR guidelines. Each data point was coded as either a score of 0 to represent that the scientific paper omitted information that should have been present, or it was given a 1 when information was provided. To assess media report translation of scientific research, we compared the data extracted from media reports with their corresponding scientific paper. For media, if the content of the report matched the information in the scientific paper, a score of 1 was assigned, and if the media report omitted or did not provide clear enough information to match the scientific paper, a score of 0 was assigned. This resulted in proportions of matching content for each domain (Multimedia Appendices 2 and 3). These proportions were used in subsequent chi-square tests.

### Balance Analysis

Assessors provided their interpretation of balance for each report and paper. Balance was defined as, *whether or not alternative explanations for findings were provided to readers*. First, balance was individually assessed at the time of the data collection and then discussed among authors. Consensus was reached with the aid of supporting quotations extracted from each resource independently. We used a multiassessor and consensus approach to validate potentially subjective assessments [25,26]. To view balance in light of the reporting quality, we averaged all domain means for each piece and compared corresponding media and scientific papers.

### Readability and Accessibility

During the data collection process, assessors extracted direct quotations to provide support for their assessments during consensus discussions. This resulted in a collection of quotations describing the *results* reported in both the media reports and scientific papers. Using these direct quotations, we assigned a Flesch-Kincaid readability score to each paper and report. The Flesch-Kincaid grade reading level indicates how difficult a passage is to comprehend based on word and sentence length; a lower score indicates an easier-to-read passage. Ideally, papers for a general audience would be written below a twelfth grade reading level [27-30]. We chose to assess readability of the Results section of the reports and papers, as this section is generally the most technically written. Inclusion of a citation to the scientific paper within media reports and the full-text accessibility of the paper was also recorded.

### Changes to Original Protocol

We had not anticipated to come across media reports with unclear details on the type of intervention administered. When this was realized, we applied the intervention exclusion criteria of the media reports to the scientific articles as well. In response to peer-review considerations, we also chose to add an additional comparative analysis using chi-square tests of proportions.

## Results

### Media Report Characteristics

Overall, 40 media reports were screened from the Google search returns. Of the 40, 17 (17/40, 43%) met inclusion criteria for this study and were retained for further analysis to referenced scientific papers (Table 1). However, many of the scientific papers referenced (11/17, 65%) could not be found with the information these media reports provided. In total, 5 of the media reports returned were on omega-3 supplementation, but they lacked the scientific rigor of peer review, so they were not included in the primary analysis. Only one of the media reports returned was clearly not based on the use of omega-3 supplements. When corresponding scientific papers were read in full, additional 6 were removed according to inclusion or exclusion criteria.

### Scientific Paper Characteristics

Table 2 provides the journals and study designs for each of the 14 scientific papers referenced by 17 media reports within this study. In total, 2 (2/14, 14%) scientific papers were referenced multiple times by different media outlets [31,32]. More than half (9/14, 64%) of the scientific papers summarized by included media reports were randomized controlled trials (RCTs; 7/9, 78%) or secondary data analyses derived from RCTs (2/9, 22%). Overall, 3 (3/14, 21%) of the papers were systematic reviews and meta-analyses or systematic reviews or meta-analyses. The remaining 2 (2/14, 14%) papers were narrative literature reviews.

The study characteristics of the scientific papers are presented in Multimedia Appendix 4. Participants varied in age from infants to those in their 80s and included both males and females located in a variety of different countries. Method of exposure to omega-3 fatty acids varied across studies from capsules to enriched products. Outcomes assessed included cognitive function, mood disorders, cardiovascular function, cancer, infant morbidity, and adult mortality.

### Primary Analysis

For our primary analysis, we calculated the proportion of parameters that were met within each reporting domain (Table 3) for each media report and scientific paper reporting on omega-3 dietary supplements (Multimedia Appendices 2 and 3). The only difference in proportions that was not statistically significant between media and scientific reporting was within the objectives domain (χ^2^_1_=0.8; *P*=.36). All other domain comparisons were significant (*P*<.05).

**Figure 2:**
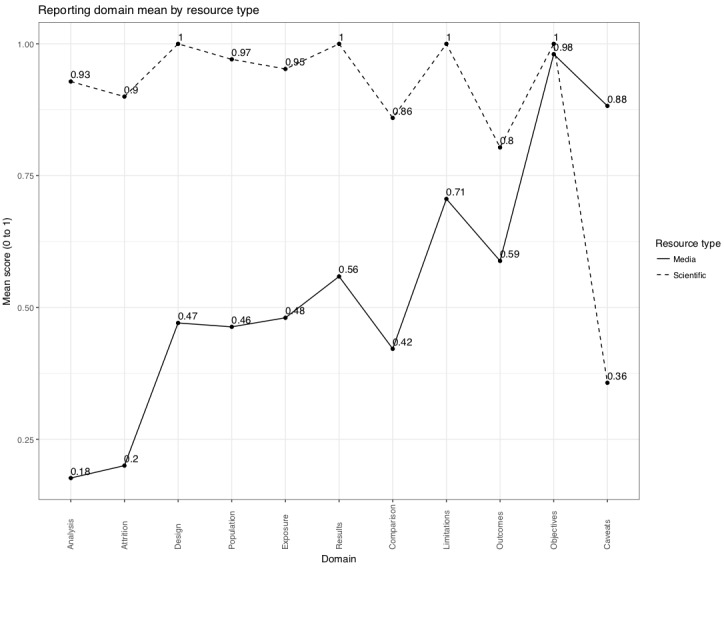
Domain mean match scores within reporting guideline domains for omega-3 dietary supplement media reports and scientific papers. Scores represent the proportion of the domain that was matched across all media reports or scientific papers. Points are connected by lines for visualization purposes.

We then generated an average domain score for all media reports and for all scientific papers (Figure 2). Scores closer to 1 signify that more parameters were met, and reporting was more complete. For each reporting domain, domain mean values for scientific papers were above 0.80, with the exception of reporting caveats mean of 0.36. Media reports show a wider variation in domain means, with study objectives mean of 0.98, limitations mean of 0.71, and caveats mean of 0.88 as the highest mean reporting scores. Media reports reviewed in this study had the lowest domain means for reporting statistical analysis with a mean of 0.18 and participant attrition with a mean of 0.20.

**Table 1:** Descriptions of all media reports obtained.

Descriptions		Type of report, n (%)															
		All media reports (N=40)		Nonsupplement (n=1)		Nonpeer-review (n=3)		Journalistic editorial or review (n=2)		Reference unclear (n=11)		Descriptive demographic report (n=2)		Whole foods (n=4)		Included reports (n=17)	
**Year of media publication**																	
	2009		10 (25)		0 (0)		1 (33)		0 (0)		3 (27)		1 (50)		1 (25)		4 (24)
	2010		10 (25)		0 (0)		0 (0)		1 (50)		5 (46)		0 (0)		1 (25)		3 (18)
	2011		10 (25)		0 (0)		2 (67)		1 (50)		2 (18)		1 (50)		2 (50)		2 (12)
	2012		10 (25)		1 (100)		0 (0)		0 (0)		1 (9)		0 (0)		0 (0)		8 (47)
**Evidence types included^a^**																	
	Evidence-based information		30 (75)		1 (100)		1 (33)		2 (100)		3 (27)		2 (100)		4 (100)		17 (100)
	Expert opinion		31 (78)		1 (100)		2 (67)		2 (100)		8 (73)		2 (100)		2 (50)		14 (82)
	Anecdotal evidence		2 (5)		0 (0)		1 (33)		0 (0)		1 (9)		0 (0)		0 (0)		0 (0)
	Journalistic opinion		6 (15)		0 (0)		1 (33)		0 (0)		3 (27)		1 (50)		0 (0)		1 (6)
**Topics discussed^a^**																	
	Effectiveness		22 (55)		1 (100)		0 (0)		2 (100)		4 (36)		2 (100)		2 (50)		11 (65)
	Quality		5 (13)		0 (0)		2 (67)		2 (100)		1 (9)		0 (0)		0 (0)		0 (0)
	Safety		8 (20)		0 (0)		2 (67)		1 (50)		4 (36)		1 (50)		0 (0)		0 (0)
	Use for multiple specific health conditions		10 (25)		0 (0)		0 (0)		2 (100)		2 (18)		0 (0)		1 (25)		5 (29)
	Use for one specific health condition		17 (43)		1 (100)		0 (0)		0 (0)		2 (18)		0 (0)		3 (75)		11 (65)
	Use for overall wellness		5 (13)		0 (0)		0 (0)		1 (50)		4 (36)		0 (0)		0 (0)		0 (0)

^a^More than one response possible for category.

**Table 2:** Scientific papers and corresponding media reports.

Journal	Media reports
*Journal of the American Medical Association* (May 2012) [31]^a^	Fish oil delivers few heart benefits, study finds, ABC News (April 2012) [33] Weighing the Evidence on Fish Oils for Heart Health, NY Times (April 2012) [34]
*Journal of the American Medical Association* (September 2012) [32]^a^	Fish oil’s heart benefits may be overstated, CNN Health (September 2012) [35] *Flawed* omega-3 meta-analysis harms public health: GOED, NutraIngredients (September 2012) [36] Questioning the Superpowers of Omega-3 in Diets, Wall Street Journal (October 2012) [37]
*Cochrane Database Systematic Reviews* (June 2012) [38]^a^	Fish Oil Fail: Omega-3s May Not Protect Brain Health After All, Time (June 2012) [39]
*Pediatrics* (September 2011) [40]^b^	Omega-3 can reduce risk of colds in babies, Telegraph UK (August 2011) [41]
*Journal of the American Medical Association* (January 2009) [42]^b^	Study: Fish Oil for Preemies May Boost Cognition, Time (January 2009) [43]
*Expert Review of Cardiovascular Therapies* (July 2009) [44]^b^	Try Fish Oil Instead of Drugs, To Your Health (February 2009) [45]
*Journal of the American Medical Association* (October 2010) [46]^b^	Fish oil doesn’t benefit new moms, babies, CNN Health (October 2010) [47]
*Journal of the American Medical Association* (November 2010) [48]^b^	Fish oil ingredient doesn’t slow Alzheimer’s, CNN Health (November 2010) [49]
*Alzheimer's & Dementia* (January 2010) [50]^b^	Fish Oil Supplements Boost Memory DHA Supplements Help Stave Off *Senior Moments*, WebMD Health News (July 2009) [51]
*Research in Developmental Disabilities* (February 2010) [52]^b^	Fallacy of fish oil revealed as study finds supplements DON’T boost children’s brain power, Daily Mail UK (April 2010) [53]
*The American Journal of Clinical Nutrition* (July 2012) [54]^c^	Omega 3 or B vitamins fail to benefit depressive symptoms, but low doses may be at fault, NeutraIngredients (June 2012) [55]
*Cancer Prevention Research* (October 2013) [56]^c^	Prostate/Prostate Cancer Nutrition/Diet Low-Fat Diet With Fish Oil Supplements Slows Growth Rate of Prostate Cancer Cells, Medical News Today (October 2011) [57]
*Journal of the American College of Cardiology* (August 2009) [58]^d^	Daily Omega-3s Recommended for Heart Benefits of Omega-3 Fatty Acids Prompt New Dosage for Heart Health, WebMD Health News (August 2009) [59]
*The Journal of Lipid Research* (August 2012) [60]^d^	Evidence is strong for omega-3’s heart health benefits: Linus Pauling Review, NutraIngredients (November 2012) [61]

^a^Systematic review or meta-analysis.

^b^Randomized controlled trial.

^c^Secondary data from a randomized controlled trial.

^d^Narrative literature review.

**Table 3:** Percent match comparisons by domain.

Domain^a^	Media (n=17), n (%)	Scientific (n=14), n (%)	χ^2^ (*df*)	*P* value
Analysis match	12 (18)	52 (93)	69.6 (1)	<.001
Attrition match	17 (20)	63 (90)	68.8 (1)	<.001
Design match	8 (47)	14 (100)	10.4 (1)	.001
Population match	61 (46)	108 (97)	69.6 (1)	<.001
Exposure match	48 (48)	80 (95)	45.9 (1)	<.001
Results match	19 (56)	28 (100)	16.3 (1)	<.001
Comparison match	40 (42)	70 (86)	31.0 (1)	<.001
Limitations match	12 (71)	14 (100)	4.9 (1)	.03
Outcomes match	39 (59)	45 (80)	5.3 (1)	.02
Objectives match	50 (98)	42 (100)	0.8 (1)	.36
Caveats match	15 (88)	5 (36)	9.3 (1)	.002

^a^Multiple domains had more than one parameter, as shown in Multimedia Appendix 2. Therefore, it was possible for a domain to have a number of matches higher than the actual sample size.

### Balance Analysis

**Figure 3:**
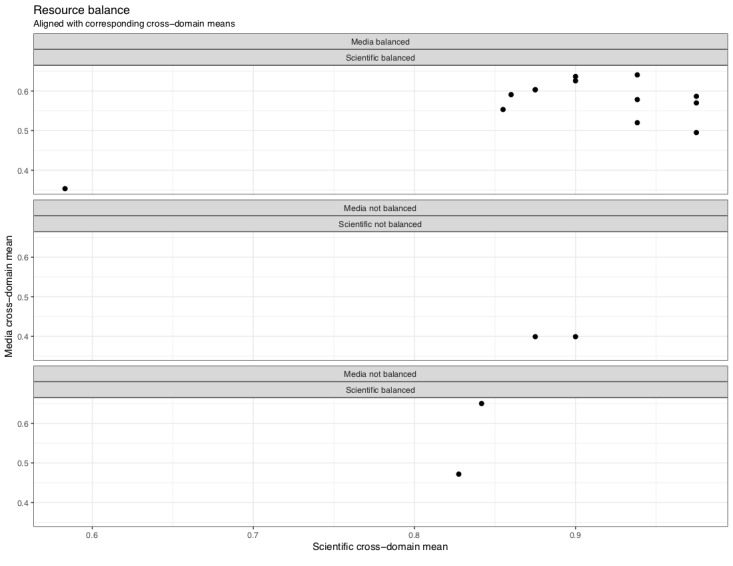
Resource balance and mean score: comparison of media report and corresponding scientific paper overall mean score, including whether or not resources were balanced in their reporting.

A total of 13 of the 17 (13/17, 76%) omega-3 dietary supplement report or paper pairings were considered to be balanced in reporting evidence (Figure 3, top panel), meaning that they presented the reader with multiple perspectives on the evidence at hand. The scientific paper that scored lowest in this group (at 0.58) was a narrative review; a corresponding media report or paper for that scored 0.35 across domain means. Although this pairing did report on multiple perspectives and therefore was found to be balanced in perspective, it contained far less detail than other report or paper pairings, including other nonsystematic reviews.

In 2 of the 17 (2/17, 12%) pairings, we considered neither media report nor scientific paper to be balanced (Figure 3, middle panel). These media reports held the lowest domain mean (0.40) and reported on RCTs. Their corresponding scientific papers scored 0.90 and 0.88, respectively, across domains. Finally, in 2 of the 17 (2/17, 12%) report or paper pairings (Figure 3, bottom panel), the scientific paper was found to be balanced, whereas the media report was assessed as unbalanced. The scientific papers that these unbalanced media reports were based on held domain means of 0.83 and 0.84. Corresponding media report domain means for these 2 pairings were 0.47 and 0.65, respectively. In none of the cases, the media report was assessed as balanced with a corresponding unbalanced scientific paper.

### Readability and Accessibility

**Figure 4:**
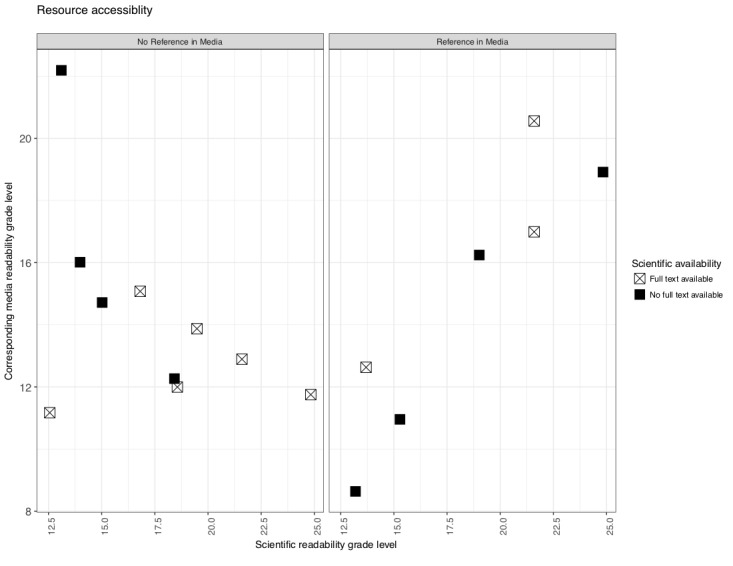
Comparison of media report and corresponding scientific paper reading grade level by whether or not a reference was provided in media report. Plot point represents whether or not scientific paper was available in full text to the general public or not.

Readability was determined through Flesch-Kincaid grade level readability scores (Figure 4). Accessibility was examined through the use of clear references in the media reports focused on omega-3 supplement, and the public availability of the scientific paper in full text. A total of 76% (13/17) of the media reports used language above a twelfth grade reading level. In addition, 59% (10/17) of the media reports did not supply a reference for the corresponding scientific paper. Moreover, 64% (9/17) of the scientific papers were not available in full text to the public. In addition, 41% (7/17) of the media reports were posted before the corresponding scientific paper was published.

## Discussion

### Principal Findings

The reporting domains where media and scientific resources converge and diverge in this study speak to the intended audience and purpose of each resource. These results suggest that more technical aspects of a study, such as statistical analysis, participant attrition, participant characteristics, and even specific study design were more frequently omitted from media reports. The intended audience for the report itself may explain these omissions. Media reports, targeting a consumer audience, reported caveats or warnings about the direct use of the supplement more frequently than scientific papers did. It is important to recognize the intentions of each type of resource, as this likely dictates consumer’s takeaway knowledge of the health information contained within.

Health information may be obtained via numerous Web-based outlets. However, not all consumers possess the capabilities to detect what is relevant or credible, nor do they necessarily always discuss the information with their physician [62,63]. Consumers have expressed mistrust in and confusion with Web-based health information in the past [18,62,64]. This confusion may be because, in part, of the format of the information presented. Media reports and scientific papers differ when it comes to the structure in which they are presented. Scientific papers are pyramidically structured, building on background, setting, and methods information, and finally presenting the results and conclusions of data analysis last. In contrast, media reports are typically presented in the opposite format. To attract the most viewers at once, media reports often present the *exciting* results and conclusions first, followed by the relevant details of the study they are representing [65]. This approach may serve to capture more readers, but there could be a detrimental trade-off in quality. If readers are given the most interesting evidence in lieu of pertinent details, the story remains incomplete. For these reasons, it was expected that media reports would contain less detailed information than the scientific papers would.

As a result of these discrepancies, both science and media may see better reader comprehension if their reporting goals were more apparent to consumers. Consumers should also be better informed on the potential dangers of using a single study to make a health care decision without input from a health care professional. One way of clarifying goals of a resource and reducing bias would be to provide balanced information. The results of our exploratory balance analysis suggest that when either science or media reported alternative viewpoints and evidence, their mean reporting score was higher than those that only reported one perspective (equating to unbalanced reporting). These findings indicate that there may be more to the relationship between reporting quality and balance overall. Including multiple viewpoints may serve to further educate consumers and instill trust in the scientific process through transparency. Consumers may not inherently notice when a piece is unbalanced or one-sided, but according to these findings, balance may be somewhat related to reporting quality, overall regardless.

When it comes to readability, we saw a stark contrast between the intended audience of media reports and scientific papers emerge. The readability of scientific papers never fell below a twelfth grade level, indicating that the information was geared toward a more educated demographic. This was to be expected, as scientific papers are intended to be read by the scientific community. It is interesting though that a majority of the media reports also scored above a twelfth grade reading level. There are limitations to the Flesch-Kincaid readability score here, which should be mentioned too. For instance, the inability to use less technical words when describing scientific principles in the Results section may have increased readability score of a media report more so than if they were reporting on a nonscientific topic.

Accessibility to scientific papers was somewhat limited for these resources. Direct references were not provided in many of the media reports (10/17, 59%). Whether that was because of the scientific paper itself not yet being published at the time the report was written or because of omission by the media report is unclear. However, the lack of reference still restricts the information consumers are able to access. Failure to include an appropriate reference to consumers places obstacles for those who want to and know they should cross-check reported findings of a study. This reduces the accessibility of scientific information for a general audience. At the time this study was run, full-text (public) availability was present for only about half of the scientific papers (n=8). Without access to a full-text paper, consumers do not have the option to verify the information within media reports if they want to bring it to their health care provider for further guidance.

### Strengths and Limitations

Prior research has taken a similar approach to ours in looking at the information presented by both press releases and corresponding print newspapers [10,12,66]. One of these studies also used Consolidated Standards of Reporting Trials reporting guidelines to grade their assessments of news reporting on press releases of RCTs, which supports the methodology that we used here [66]. These studies align with our findings in the sense that the scientific research in many ways relates to the corresponding media report, whether it is in the tone, quality, or overall coverage. Another similar paper compared print media with scientific guidelines as well [13], but to our knowledge, this is the first study to view quality comparisons between *Web-based* news media and corresponding health research. It is certainly the first to focus on this popular health supplement as a topic of interest. One weakness in comparing our work with prior literature was that in the aforementioned studies, which viewed scientific claims made in print media, researchers found a tendency for media reports to under-report limitations and overlook potential risks [25,26]. We found nearly the opposite, in that limitations were fairly well represented in the media reports (mean=0.71), and these sources provided risks, warnings, or caveats for consumers more frequently than the corresponding scientific papers did. This may be dependent on the type of scientific reporting.

The largest limitation in this study was the small sample of papers that could actually be compared. A 4-year retrospective sample consisting of 40 media reports is by no means comprehensive for all of the literature available. Knowing this, we sought to find the papers that would be considered the *most* influential to increased omega-3 supplement use trends. Google’s PageRank Algorithm sifts the most influential returns to the top of the news search [16,17]. By choosing the top 10 returns in our stratified convenience sample, we were selecting the returns that most users were likely to have had exposure to, and therefore, influence from [16-23,67]. Several media reports were initially excluded (n=11), simply because they lacked the necessary information to clearly find a corresponding scientific paper. This was a large limitation to the number of papers and reports that could be compared with one another. It does also demonstrate another issue that health information consumers might experience with regard to accessibility though. Even if consumers know to cross-check a media report for missing information, there is no way for them to accurately do so when they cannot identify the scientific paper.

Further sample size restrictions were made when papers or reports failed to meet inclusion criteria. This inclusion criterion was set in place to examine only those report or paper combinations that were specifically about the effects of omega-3 dietary supplementation on health. We had expected that some papers may be vague on details of an intervention, and perhaps some might mention supplementation and dietary changes together. What we had not expected to see were vague media reports about whether or not participants had been given a supplement or dietary intervention (n=4). In these instances, it was not until we reviewed the scientific paper that we were able to discern whether or not the pairing fits inclusion criteria. Providing consumers with vague information on the type of intervention used is potentially misleading, especially considering that these consumers might make health care decisions based off of it. Further work in this area of study could be improved with alterations to the inclusion and exclusion criteria that would allow for more papers to be obtained for the comparison. This might include incorporating more dietary supplements or even incorporating more study designs.

A strength of this study was that it was focused on Web-based news media, rather than print media, which appears to be the primary focus of much of the prior work in this field [10,12,13,25,26,66]. Previous studies have indicated that there is a difference in the information presented by these 2 forms of media reporting, in that Web-based media may present a wider variety of themes when a scientific topic is searched [20]. This could also be seen as a drawback because different demographic groups may be more or less likely to obtain their information from print versus Web-based media sources. Therefore, the applicability of these results may be limited to those who tend to use Web-based news media resources.

The final limitation of this study was the use of somewhat subjective assessments of balance. Although the quality of reporting and accessibility could be determined by either a match of information or well-known readability scoring, balance is a bit more nuanced. The assessors did, however, individually decide how balanced they felt each piece was, and they were also required to support their judgment with specific examples from the resource to come to a consensus. To decrease subjectivity further, assessors were provided with specific definitions of *balance* for assessing it in a piece. Future work could include a deeper look into the ways that balance the impacts of the health information consumer’s interpretation and application of Web media information to their health care decision making.

## Appendix

Multimedia Appendix 1 Guidelines used for data extraction: parameters collected under each reporting domain in this study according to respective EQUATOR reporting guideline’s item numbers.

Multimedia Appendix 2 Percent of scientific resources matching. This figure illustrates the percent of the sample where scientific articles matched to the appropriate EQUATOR guideline.

Multimedia Appendix 3 Percent of media resources matching. This figure illustrates the percent of the sample where media reports matched to their corresponding scientific article. If the scientific article did not contain relevant information, media reports also not containing the information were marked as "matched;" therefore, media reports could only be held accountable for reporting what scientific articles presented.

Multimedia Appendix 4 Descriptive characteristics of peer-reviewed scientific articles included in this analysis.

## Abbreviations

ALA: alpha-linolenic acid
NHIS: National Health Interview Survey
PUFA: polyunsaturated fatty acids
RCT: randomized controlled trial
