# Supplementary material for: Health Information Discrepancies Between Internet Media and Scientific Papers Reporting on Omega-3 Supplement Research: Comparative Analysis
Source: Interact J Med Res. 2018 Oct 1;7(2):e15. doi: 10.2196/ijmr.8981 (PMC6231791; doi:10.2196/ijmr.8981)
Supplement: Multimedia Appendix 1 [file ijmr_v7i2e15_app1.pdf]

Multimedia Appendix

Reporting Domain	CONSORT Item No.	STROBE Item No.	PRISMA Item No.	Description of Domain Parameters
**Objective**	2	3	4	Presented study objectives, independent and dependent variables.
**Design**	1, 3	1, 4	1	Present key elements of study design.
**Participants**	4	6	6	Give the eligibility including diagnostic criteria, species, age, gender, and the sources and methods of selection of participants.
**Exposure and Comparison**	5, 11	7	6	Clearly define all outcomes and exposures/interventions, including the type, frequency, dose, setting, and timing of administration with enough details to allow for replication.
**Outcomes**	6	8	11	For each outcome variable, give sources of data, setting, and details of methods of assessment (measurement).
**Analysis**	12	12	12-16	Describe all statistical methods used to examine comparisons, including adjustments to control for confounding. Explain how missing data were addressed.
**Attrition**	13	13	17	Report numbers of individuals at each stage of the study.
**Results**	14-19	14-17	18-23	For all outcomes give unadjusted estimates, confounder-adjusted estimates, results for each group, estimated effect sizes, and precision where applicable. Make sure that any adjustments are clear to the reader.
**Limitations**	20	19	25	Discuss limitations of the study, taking into account sources of potential bias or imprecision.
**Caveats (for direct clinical application)**	21, 22	20, 21	24	Provide an interpretation of the evidence considering direct clinical applicability within the context of generalizability.
