# Supplementary figures and images for: Health Information Discrepancies Between Internet Media and Scientific Papers Reporting on Omega-3 Supplement Research: Comparative Analysis
Source: Interact J Med Res. 2018 Oct 1;7(2):e15. doi: 10.2196/ijmr.8981 (PMC6231791; doi:10.2196/ijmr.8981)

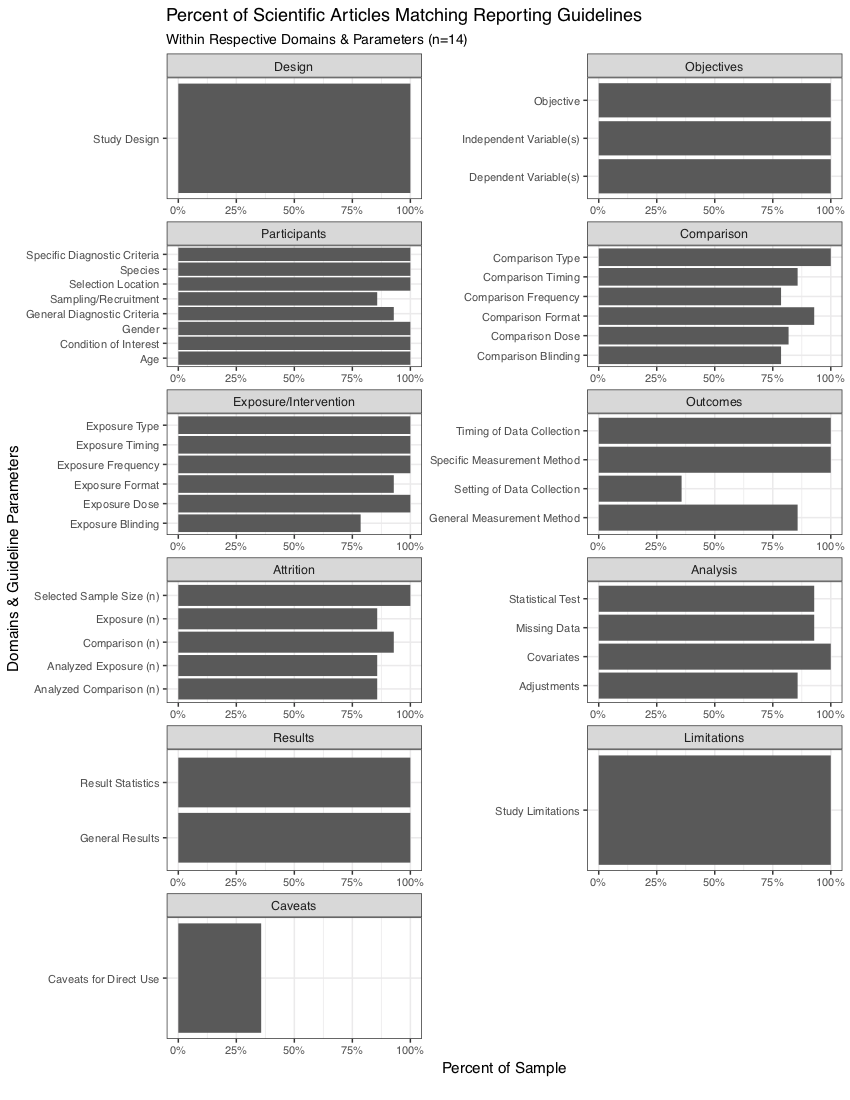

Supplement: Multimedia Appendix 2 [file ijmr_v7i2e15_app2.png]

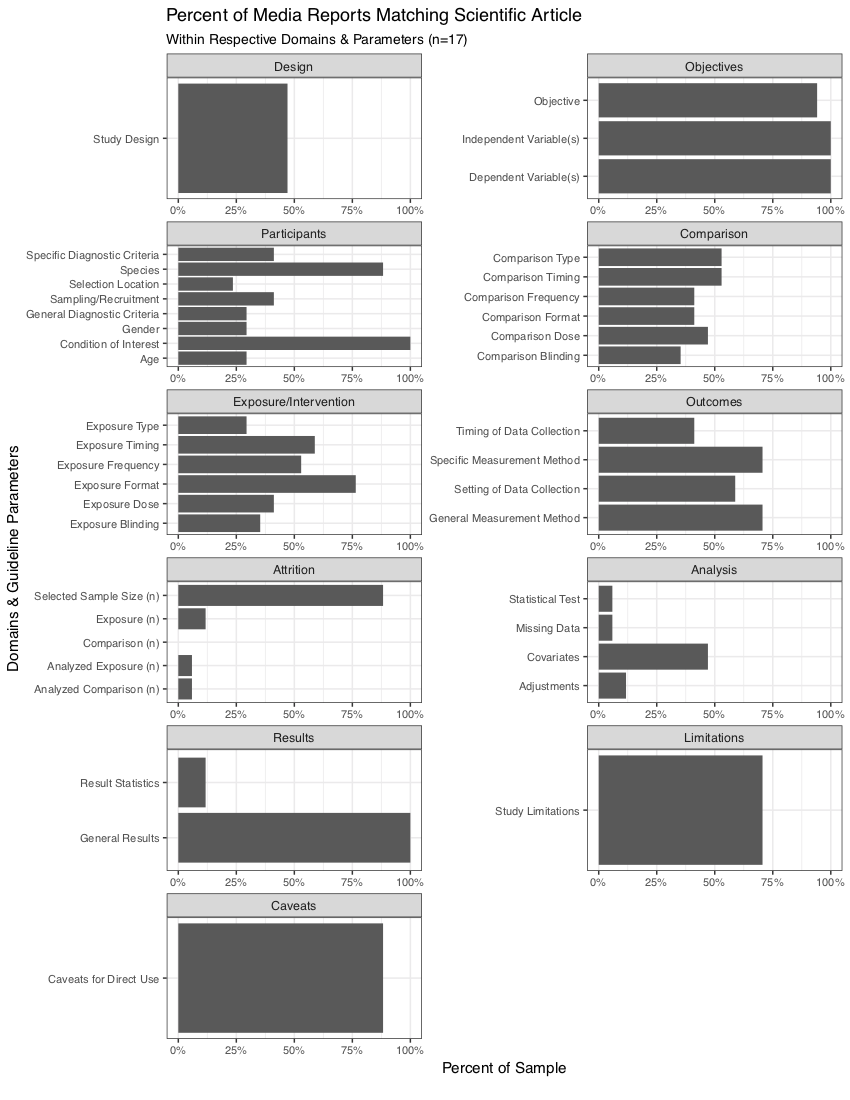

Supplement: Multimedia Appendix 3 [file ijmr_v7i2e15_app3.png]
