# Supplementary material for: Health Information Discrepancies Between Internet Media and Scientific Papers Reporting on Omega-3 Supplement Research: Comparative Analysis
Source: Interact J Med Res. 2018 Oct 1;7(2):e15. doi: 10.2196/ijmr.8981 (PMC6231791; doi:10.2196/ijmr.8981)
Supplement: Multimedia Appendix 4 [file ijmr_v7i2e15_app4.pdf]

Multimedia Appendix

Scientific Publication	Population	Exposure	Comparison	Outcome Measures
Journal of the American Medical Association (May 2012) [31]	M/F 40-80 years, with pre-existing cardiovascular dysfunction. Located in the US, Asia, Europe.	O3FAS with EPA or DHA from 0.4 to 4.8 g/d, for at least 1 year.	Oil based placebo	Occurrence of CV events or unscheduled CV interventions.
Journal of the American Medical Association (Sep 2012) [32]	M/F 49-70 years, requiring primary or secondary prevention of CVD. Located in the US, Europe, Asia.	O3FAS (1.51 g) with 0.77g/d EPA & 0.60g/d, DHA, for a duration of 1-6.2 years.	Control diet or Oil based placebo	All-cause mortality, or CV event.
Cochrane Database Systematic Reviews (Jun 2012) [33]	M/F > 59, without pre-existing dementia or cognitive impairment. Located in the Netherlands, England & Wales.	O3FAS containing 400 mg - 1800 mg/d EPA/DHA for a duration of 26 weeks, 24 months, or 40 months.	Oil based placebo	Cognitive function test scores.
Pediatrics (Sep 2011) [34]	F aged 18 to 35 years, in gestation weeks 18 to 22 and offspring. Located in Mexico.	Women received 200 mg DHA capsules 2x/d, starting between gestation weeks 18-22 until parturition.	Oil based placebo	Maternal reporting of cold/flu symptoms.
Journal of the American Medical Association (Jan 2009) [35]	M/F infants born before 33 weeks' gestation. Located in Australia.	Breastmilk of infants mothers, who were given six 500-mg DHA-rich tuna oil capsules per day.	Oil based placebo	Developmental test scores.
Expert Review of Cardiovascular Therapies (Jul 2009) [36]	M/F > 18 years, with clinical evidence of heart disease. Located in Italy.	Daily 1g O3FAS (850-882 mg EPA &DHA) plus 10 mg rosuvastatin for 1 year.	Placebo	Time to death or CV event.
Journal of the American Medical Association (Oct 2010) [37]	Mothers < 21 weeks gestation Located in Australia. Follow up with their offspring (M/F) at 18 months old.	Mothers took 800 mg/d of DHA and 100 mg/d of EPA from study entry until birth of their child.	Oil based placebo	Depressive symptom scores (mothers). Cognitive and language development scales (offspring).
Journal of the American Medical Association (Nov 2010) [38]	M/F, mean age of 76(8.7 SD) with probable Alzheimer's disease. Located in U.S.	1g DHA capsules 2x/d, for 18 months.	Oil based placebo	Cognitive and functional test scores.

Scientific Publication	Population	Exposure	Comparison	Outcome Measures
Alzheimer's & Dementia (Jan 2010) [39]	M/F > 55 years, with memory and mental state examination scores meeting cutoff points. Located in the United States.	O3FAS containing 300 mg/d DHA (40% DHA, 15% EPA), plus antioxidants, for 24 weeks.	Oil based placebo	Visuospatial learning and episodic memory function test scores.
Research in Developmental Disabilities (Feb 2010) [40]	M/F, 8-9 years old, without omega-3 PUFA supplementation in the past 6 months. Located in Wales, UK.	O3FAS containing 200mg DHA and 28mg EPA, plus Vitamins A, C, D, and E 2x/d for 16 weeks.	Oil based placebo	Learning and behavior test scores.
The American Journal of Clinical Nutrition (Jul 2012) [41]	M/F aged 45-80, with reported myocardial infarction, unstable angina, or ischemic stroke within the preceding 12 months. Located in France.	O3FAS containing B vitamins, 600 mg EPA and DHA in a 2:1 ratio, 2x/d for one year.	B-vitamins or placebo	Depressive symptom scale scores.
Cancer Prevention Research (Jan 2014) [42]	M, mean age of 60, diagnosed with prostate cancer. Located in Los Angeles, CA.	O3FAS with 5 grams of fish oil per day, plus Low-fat diet (20% Kcal fat) for an average of 28 to 30 days.	Western diet, no O3FAS	Prostate tissue and serum sample lab values, and cell cycle progression score.
Journal of the American College of Cardiology (Aug 2009) [43]	M/F of varied ages in primary prevention post myocardial infarction following heart failure. Located in multiple countries, including U.S.	Intervention studies: O3FAS with 460mg-5.1g/d EPA and DHA, plus usual care for 12 weeks to 13 years.	Intervention studies: placebo	Occurrence of CV events, death, arterial changes, and heart rate variability improvement.
The Journal of Lipid Research (Dec 2012) [44]	M/F age 20-70; some participants having CVD and some healthy. Located in the US, Italy, Japan, Sweden, and Greenland.	Intervention studies: O3FAS with 300-2000mg DHA and EPA for various time periods.	Intervention studies: vitamin E, Oil based placebo, statins, or vitamin B	Various CV risk factors were measured across studies.
**Abbreviations.** O3FAS: Omega-3 Fatty Acid Supplement; EPA: Eicosapentaenoic Acid; DHA: Docosahexaenoic Acid; CV/CVD: Cardiovascular (CV) Disease				
